# Preventing Zoonotic Diseases in Immunocompromised Persons: The Role of Physicians and Veterinarians

**DOI:** 10.3201/eid0602.000219

**Published:** 2000

**Authors:** Norbert Nowotny, Armin Deutz

**Affiliations:** * Institute of Virology, University of Veterinary Sciences, Vienna, Austria; and †Animal Health Service, Styrian Provincial Government, Department of Veterinary Affairs, Graz, Austria

**Keywords:** coxiella burnetii, brucella sp, chlamydia psittaci, leptospira sp, toxoplasma gondii, toxocara canni/cati, Austria,


### Preventing Zoonotic Diseases in Immunocompromised Persons: The Role of Physicians and Veterinarians

**To the Editor:** We read with great interest the article by Grant and Olsen on the role of physicians and veterinarians in preventing zoonotic diseases in immunocompromised persons (1) and the letter by Barton et al. on the risk of pregnant women and young infants for pet-associated illnesses (2). Essentially all aspects and conclusions of the study by Grant and Olsen are also valid in Europe.

In Austria, veterinarians are well educated in zoonotic infections. However, it is impossible for practitioners to know all zoonotic agents in detail. In addition to immunocompromised persons, pregnant women, and young infants, persons in certain occupations are at higher risk for zoonotic infections. Veterinarians are one of these groups. We have completed seroepidemiologic studies involving veterinarians (3) and are testing other groups at high risk, such as slaughterhouse workers, farmers, and zoo employees.

We surveyed 52% of the veterinarians in an Austrian federal state who agreed to participate in the study. They completed a questionnaire and provided case histories so that risk factors could be assessed. We also obtained blood samples. The sera were tested for antibodies to viral, bacterial, and parasitic zoonotic agents. After correlating the serologic results with the statements in the questionnaire, a statistical analysis, and another questionnaire of selected participants, we found transmission of zoonotic agents from animals to veterinarians for influenza A virus H1N1 (prevalence of the infection was much higher among veterinarians than in the general population; a significant number of seropositive veterinarians were swine practitioners [chi-square = $p^{xx} \leq .01$]); *Coxiella burnetii* (veterinarians who removed bovine placenta without gloves had a higher risk [chi-square = $p^{xxx} \leq .001$] of acquiring *Coxiella burnetii* infections); *Brucella* sp.; *Chlamydia psittaci; Leptospira* sp.; *Toxoplasma gondii;* and *Toxocara canis/cati* (antibody prevalence was 20 times higher among veterinarians than in the general Austrian population). As a result of the survey, veterinarians know about their profession-specific risk factors and take adequate measures to prevent infections; also, they are more qualified to advise pet owners and persons of other professions at high risk.

In addition to the pathogens listed by Grant and Olsen and the agents listed in this letter, some other zoonotic agents present (at least in Central Europe) are cowpox (mainly acquired from cats; 4-6) and parapox viruses, lymphocytic choriomeningitis virus, Newcastle disease virus, *Erysipelothrix rhusiopathiae,* and *Capillaria hepatica* (7). On the other hand, animals are not the source of human disease such as leukemia; there is no evidence that feline leukemia virus and feline immunodeficiency virus, for example, are transmitted to humans (8). The risk of acquiring a zoonotic infection from a pet animal is definitely lower than the emotional and health benefits of pet ownership.

**Norbert Nowotny* and Armin Deutz†**
*Institute of Virology, University of Veterinary Sciences, Vienna, Austria; and †Animal Health Service, Styrian Provincial Government, Department of Veterinary Affairs, Graz, Austria

### References

1. Grant S, Olsen CW. Preventing zoonotic diseases in immunocompromised persons: the role of physicians and veterinarians. Emerg Infect Dis 1999;5:159-63.
2. Barton LL, Villar RG, Connick M. Pet-associated zoonoses. Emerg Infect Dis 1999;5:598.
3. Nowotny N, Deutz A, Fuchs K, Schuller W, Hinterdorfer F, Auer H, et al. Prevalence of swine influenza and other viral, bacterial, and parasitic zoonoses in veterinarians. J Infect Dis 1997;176:1414-5.
4. Nowotny N. The domestic cat: a possible transmitter of viruses from rodents to man. Lancet 1994;343:921.
5. Nowotny N. Serologische Untersuchungen von Hauskatzen auf potentiell humanpathogene Virusinfektionen wildlebender Nagetiere. Zentralblatt für Hygiene und Umweltmedizin 1996;198:452-61.
6. Nowotny N, Fischer OW, Schilcher F, Schwendenwein I, Loupal G, Schwarzmann Th, et al. Pockenvirusinfektionen bei Hauskatzen: klinische, pathohistologische, virologische und epizootiologische Untersuchungen. Wiener Tierärztliche Monatsschrift 1994;81:362-9.
7. Juncker-Voss M, Prosl H, Lussy H, Enzenberg U, Auer H, Nowotny N. Serological detection of *Capillaria hepatica* by indirect immunofluorescence assay. J Clin Microbiol 2000;38:431-3.
8. Nowotny N, Uthman A, Haas OA, Borkhardt A, Lechner K, Egberink HF, et al. Is it possible to catch leukaemia from a cat? Lancet 1995;346:252-3.

To the Editor: We read with great interest the article by Grant and Olsen on the role of physicians and veterinarians in preventing zoonotic diseases in immunocompromised persons (1) and the letter by Barton et al. on the risk of pregnant women and young infants for pet-associated illnesses (2). Essentially all aspects and conclusions of the study by Grant and Olsen are also valid in Europe.

In Austria, veterinarians are well educated in zoonotic infections. However, it is impossible for practitioners to know all zoonotic agents in detail. In addition to immunocompromised persons, pregnant women, and young infants, persons in certain occupations are at higher risk for zoonotic infections. Veterinarians are one of these groups. We have completed seroepidemiologic studies involving veterinarians (3) and are testing other groups at high risk, such as slaughterhouse workers, farmers, and zoo employees.

We surveyed 52% of the veterinarians in an Austrian federal state who agreed to participate in the study. They completed a questionnaire and provided case histories so that risk factors could be assessed. We also obtained blood samples. The sera were tested for antibodies to viral, bacterial, and parasitic zoonotic agents. After correlating the serologic results with the statements in the questionnaire, a statistical analysis, and another questionnaire of selected participants, we found transmission of zoonotic agents from animals to veterinarians for influenza A virus H1N1 (prevalence of the infection was much higher among veterinarians than in the general population; a significant number of seropositive veterinarians were swine practitioners [chi-square = p^xx^ .01]); *Coxiella burnetii* (veterinarians who removed bovine placenta without gloves had a higher risk [chi-square = p^xxx^ .001] of acquiring *Coxiella burnetii* infections); *Brucella* sp.*; Chlamydia psittaci; Leptospira* sp.; *Toxoplasma gondii;* and *Toxocara canis/cati* (antibody prevalence was 20 times higher among veterinarians than in the general Austrian population). As a result of the survey, veterinarians know about their profession-specific risk factors and take adequate measures to prevent infections; also, they are more qualified to advise pet owners and persons of other professions at high risk.

In addition to the pathogens listed by Grant and Olsen and the agents listed in this letter, some other zoonotic agents present (at least in Central Europe) are cowpox (mainly acquired from cats; 4-6) and parapox viruses, lymphocytic choriomeningitis virus, Newcastle disease virus, *Erysipelothrix rhusiopathiae,* and *Capillaria hepatica* (7). On the other hand, animals are not the source of human disease such as leukemia; there is no evidence that feline leukemia virus and feline immunodeficiency virus, for example, are transmitted to humans (8). The risk of acquiring a zoonotic infection from a pet animal is definitely lower than the emotional and health benefits of pet ownership.
